# Safety and efficacy of two techniques of temporary ovarian suspension to the anterior abdominal wall after operative laparoscopy

**Published:** 2018-06

**Authors:** Omar M Abuzeid, John Hebert, Mohammad Ashraf, Mohamed Mitwally, Michael P Diamond, Mostafa I Abuzeid

**Affiliations:** Departmentof OB/GYN, Hurley Medical Center, Michigan State University College of Human Medicine, Flint Campus, Flint, Michigan, USA; Division of Reproductive Endocrinology and Infertility, Hurley Medical Center, Michigan State University College of Human Medicine, Flint Campus, Flint, Michigan, USA; IVF Michigan Rochester Hills & Flint, PC, Rochester Hills, Michigan, United States; San Antonio Reproductive Associates, San Antonio, Texas, USA; Department of OB/GYN, Augusta University, Augusta, Georgia, USA

**Keywords:** abdominal wall, absorbable suture, non-absorbable suture, ovarian suspension, safety and efficacy

## Abstract

**Background:**

This retrospective study compares the safety and efficacy of temporary ovarian suspension (TOS) to the anterior abdominal wall using absorbable versus non-absorbable suture after operative laparoscopy to elevate the ovaries away from the ovarian fossa to reduce postoperative adhesion development.

**Methods:**

Patients (n=152) underwent TOS to the anterior abdominal wall at the conclusion of surgery between 1998 and 2017. One hundred forty-two patients underwent operative laparoscopy for advanced stages of endometriosis (93.4%) and 10 patients for other indications (6.6%). In 78 patients the ovaries were suspended to the fascia using absorbable 3-0 plain catgut sutures (Group 1). In 74 earlier patients non-absorbable 3-0 mono-lamentous nylon was used to suspend the ovaries to the anterior abdominal (Group 2).

**Results:**

In both groups there was no reported incidence of any major intra-operative complications such as bleeding, or late complications such as infection, hematoma or bowel herniation through the suture loop and its sequalae (bowel obstruction or strangulation). In all patients in both groups the ovaries were present in its anatomical location on transvaginal ultrasound scan, one week after surgery following absorption or removal of the TOS suture. There was no significant difference in clinical pregnancy (34.3% vs 44.2%) and delivery (31.3% vs 36.5%) rates in patients who conceived with non-IVF methods between Group 1 and Group 2 respectively.

**Conclusions:**

TOS to the anterior abdominal wall, using absorbable or non-absorbable sutures, in an attempt to reduce postoperative adhesion development between the ovary and ovarian fossa, is simple, safe, easy to learn, and has potential effectiveness.

## Introduction

Pelvic adhesions can result from endometriosis, pelvic inflammatory disease and previous surgeries. Post-operatively the ovaries and the pouch of Douglas are the most common areas for adhesions formation (Diamond, 1991). Advanced stages of endometriosis are usually associated with adhesions of the ovaries to the ovarian fossae and peri-tubal and peri-ovarian adhesions. Pelvic adhesions can cause chronic pelvic pain, dyspareunia and intestinal obstruction (DiZerega, 1994). In addition, such adhesions have a major impact on the fertility potential of female patients as a result of mechanical factors of infertility. Operative laparoscopy for advanced endometriosis has failed to prevent post-operative adhesion formation with a reported incidence in 50%-100% of such patients (Canis et al., 1992; DiZerega, 1994). Prevention of pelvic adhesions by a variety of strategies has been attempted over the years. Post-operative adhesion can be reduced by adopting a good surgical technique, minimizing peritoneal injury and meticulous hemostasis during operative laparoscopy. However, post-operative adhesion development continues to occur at sites throughout the pelvis even after the use of anti- adhesive agents such as Interceed, Seprafilm and ADEPT solution (Hawthorn et al., 2004; Rajab et al., 2010; Ten Broek et al., 2014; Ahmad et al., 2015).

Ovarian suspension techniques have been proposed after ovariolysis and excision of endometrioma to reduce occurrence and recurrence of adhesions. Over the last 15 years many groups published their experience with temporary ovarian suspension (TOS) for various indications and by various techniques (Lee et al., 1995; Quahba et al., 2004; Mitwally et al., 2006; Chapman et al., 2007; Carbonnel et al., 2011; Poncelet et al., 2012; Pellicano et al., 2014; Pergialiotis et al., 2016). More recently, Abuzeid O et al. (2018) published a video describing a modified technique to temporary suspend the ovary to the fascia of the anterior abdominal wall using an absorbable suture with 3-0 plain catgut (Abuzeid O et al., 2018). The purpose of this study is to compare the safety and efficacy of TOS to the anterior abdominal wall using absorbable suture with 3-0 plain catgut to non-absorbable mono-filaments nylon suture after operative laparoscopy.

## Materials and methods

This retrospective cohort study included 152 patients who underwent TOS to the anterior abdominal wall between 1998 and 2017 and received an exemption from the oversight of the Hurley Medical Center Institutional Review Board (IRB). The majority of patients presented with infertility (95.6%) and had advanced endometriosis (93.4%) [American Society for Reproductive Medicine 2007]. Seven patients (4.6%) consulted for the evaluation of persistent ovarian cysts. Work-up of infertility included: complete semen analysis, hysterosalpingogram, trans-vaginal (TV) 2D ultrasound scan (US) and TV 3D US (since 2008) with saline infusion sonohysterogram (SIH), hormonal profile including serum TSH, prolactin, day 3 FSH and LH levels, Anti-Mullerian hormone (since 2013) and laparoscopy and hysteroscopy when indicated.

Patients’ demographic data, detailed surgical procedures and any intra-operative or post-operative complications and reproductive outcome was extracted. The population studied was divided into two groups depending on the technique used to suspend the ovaries. The study group (Group 1) included 78 patients who had their ovaries temporary suspended using absorbable 3-0 plain catgut suture between 2011 and 2017. The control group (Group 2) included 74 patients who had the ovaries temporary suspended using non-absorbable 3-0 mono-filaments nylon suture between 1998 and 2010.

A video operative laparoscopy equipment with a four-portal entry technique was performed to allow for maximum access and maneuverability of instruments (Abuzeid O et al., 2018). During operative laparoscopy, unilateral or bilateral excision of endometriomas and / or ovariolysis was performed as previously described (Raju et al., 2015; Abuzeid O et al., 2018). In addition, one or more of the following procedures was performed as needed: salpingolysis, fimbrioplasty, salpingostomy, and argon beam coagulation or CO2 laser vaporization of endometriotic implants or small endometriomas (Abuzeid M et al., 2009; Mitwally et al., 2009). In all patients, TOS (unilateral or bilateral) was performed in an attempt to separate adhesiogenic surfaces, during the immediate 3-5 days’ time period after a surgical procedure, during which time the surface was remesotheliazed either with or without adhesions.

### Technique of ovarian suspension in Group 1:

**Figure 1:**
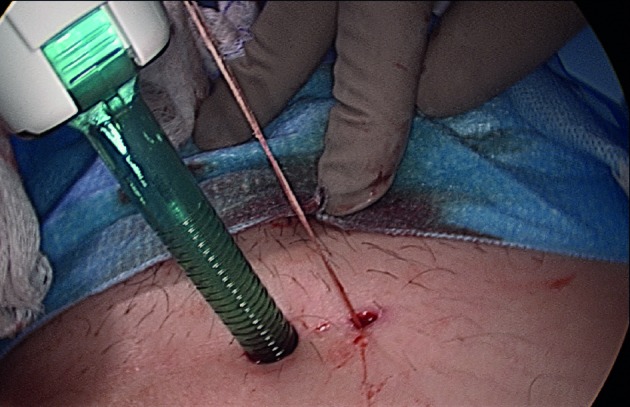
This picture illustrates the 3 mm skin incision through which one end of 3-0 plain catgut suture was brought out using endoclose device. The device was re-introduced through the same incision and the other end of the suture was brought out before tying the suture above the fascia.

**Figure 2:**
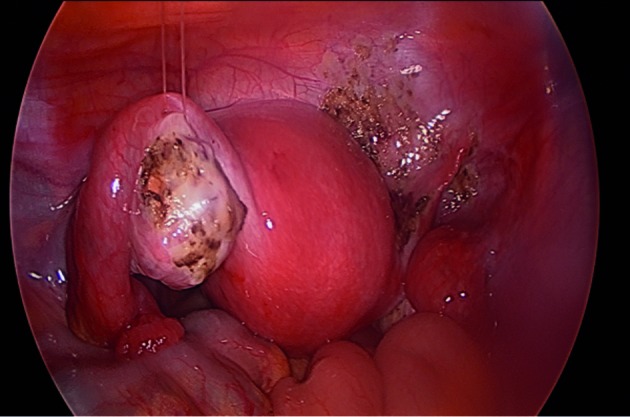
left ovary is suspended to fascia of the anterior abdominal wall using non-absorbable 3-0 plain catgut.

An absorbable 3-0 plain catgut suture (ACE Surgical Supply Co, Inc., Brockton, MA, USA) was used to elevate the ovary away from the ovarian fossa towards the abdominal wall. The ends of the sutures were brought out of the peritoneal cavity through a 3-mm skin incision using Endo-Close device (Medtronic, Minneapolis, MN, USA) or a Carter- Thomason device (Carter-Thomason Close Sure System, Cooper Surgical, Inc, Trumbull, CT, USA) [Figure 1]. Care was taken to ensure that the points of entry and exit of the suture through the fascia were very close to each other. The suture was tied over the fascia while allowing CO2 gas out of the peritoneal cavity to ensure that the suture remained under tension and the ovary suspended well without touching the abdominal wall. Re-insufflation was performed to insure the position of the ovary (Figure 2). The procedure was terminated after leaving 1 liter of Lactated Ringer’s solution (LR) or 500 cc of ADEPT solution (Baxter Healthcare Corp, Deerfield, IL, USA) in the peritoneal cavity for hydro-flotation. The skin incision for the suspension area was approximated using steri-strips.

### Technique of Ovarian Suspension in Group 2:

The TOS procedure was performed using non- absorbable mono-filaments nylon suture (U.S. Surgical, Norwalk, CT, USA), that required removal after 5-7 days, as described before (Abuzeid M et al., 2002).

Patients were discharged home the same day when they were alert, stable, voiding without difficulty, tolerating food without nausea or vomiting and tolerating pain on oral pain medication. Oral narcotic pain medication, or nonsteroidal anti-inflammatory tablets were used postoperatively as needed. During the first week, patients were instructed to take their temperature every 8 hours while awake, report readings above 100.4° F, any problems during urination, and (up to 8-weeks post-operative) any other complication. In patients in Group 1 the ovaries were found in its anatomical location on TV US that was done one week after surgery, while in Group 2 this was done 5-7 days post-operatively. Infertile patients were counselled regarding the best treatment option to enhance their chances of conceiving. Some patients were elected to try to conceive on their own for 6-12 months. Patients with ovulatory disorders were treated with Clomiphene Citrate or Aromatase inhibitor. Additional methods to boost the chances, such as controlled ovarian stimulation (COS) with or without Intra-Uterine Insemination (IUI) or In-Vitro Fertilization and Embryo Transfer (IVF-ET) were offered as indicated. Ovarian reserve tests such as antral follicle count and day 3 serum FSH levels were done in patients who underwent COS + IUI or IVF-ET. Sixteen patients in Group 1 were excluded from pregnancy outcome analysis (four patients were not trying to conceive and 12 patients had their surgery less than one year from the date of conclusion of the study and did not have IVF-ET during the same period of time). Only one patient in Group 2 was excluded from pregnancy outcome analysis as she was not trying to conceive. Pregnancy outcome after non-IVF methods was calculated in both groups after excluding patients with husbands who had severe male factor infertility that required IVF-ET using Intra-Cytoplasmic Sperm Injection (ICSI) procedure (four patients in each group). In addition, pregnancy outcome after non-IVF-ET methods was re-calculated after excluding 43 patients (26 in Group 1 and 17 in Group 2) who conceived with IVF-ET treatment during the duration of the study.

The primary outcome measure of this study was the safety of TOS procedure determined by the incidence of intra-operative, immediate, and late (up to 8 weeks) complications of surgery. The secondary outcome measures were the efficacy of TOS procedure determined by the anatomical location of the ovaries one week after surgery and by its reproductive outcome. The follow-up period was up to 2 years. Statistical analysis was performed using Student Paired T-Test and Chi-Square analysis where appropriate.

## Results

The majority of patients (142) had endometriosis (93.4%), 61.3% had stage 3 endometriosis and 38.7% had stage 4 endometriosis. The remaining ten patients (6.6%) had other pathologies, seven patients had ovarian cysts for which they underwent ovarian cystectomy and three patients had ovariolysis for adhesions related to pelvic inflammatory disease. Table 1 summarizes the demographic data of the population. Table 2 illustrates a comparison of the different procedures that were performed in each group.

**Table I:** Demographic Data

		Group 1 No.=78	Group 2 No.=74	P value	Total population No.=152
Age (Years)		31.9 ± 5.0	32.1 ± 5.2	NS	32.0 ± 5.1
Duration of Infertility (Years)		2.9 ± 2.0	2.6 ± 1.9	NS	2.7 ± 1.9
Day 3 FSH (mIU/mL)		7.4 ± 3.2	6.3 ± 2.4	NS	7.2 ± 3.1
% Primary Infertility		49.3%	67.6%	0.027	58.5%
% History of Miscarriage		27.0%	12.1%	0.034	20.5%
% Male Infertility		38.7%	20.5%	0.016	29.7%
% Ovulatory Disorder		16.0%	21.9%	NS	18.9%
% Tubal Factors		46.7%	12.5%	0.000	29.9%
% Endometriosis		89.7%	97.3%	NS	93.4%
	% stage 3	61.5%	52.7%	NS	57.9%
	% stage 4	28.2%	44.5%	0.036	42.1%
% Uterine factors *		59.0%	29.7%	0.000	44.7%
	% Incomplete septum/Significant arcuate uterine anomaly	53.8%	24.3%	0.000	39.5%
	% Myomectomy	2.6%	5.4%	NS	3.9%
	% Polypectomy	10.4%	0.0%	0.004	5.3%

* All uterine factors were corrected at time of surgery

**Table II:** Procedures performed during Operative Laparoscopy in each Group and total population.

		Group 1 No.=78	Group 2 No.=74	P value	Total population No.=152
# Patients with endometriosis (%)		70 (89.7%)	72 (97.3%)	NS	142 (93.4%)
# patients with endometriosis ablation (%)		70 (89.7%)	72 (97.3%)	NS	142 (93.4%)
# patients with endometrioma ablation (%)					
	Unilateral	5 (6.4%)	1 (1.4%)	NS	6 (3.9%)
	Bilateral	1 (1.3%)	0 (0%)	NS	1 (0.7%)
# patients with endometrioma Excision (%)					
	Unilateral	25 (32.1%)	14 (18.9%)	0.064	39 (25.7%)
	Bilateral	0 (0.0%)	6 (8.1%)	0.010	6 (3.9%)
# patients with ovariolysis (%)					
	Unilateral	37 (48.1%)	30 (49.2%)	NS	67 (48.6%)
	Bilateral	33 (42.9%)	21 (34.4%)	NS	54 (39.1%)
# patients with salpingolysis (%)					
	Unilateral	26 (33.3%)	9 (12.2%)	0.002	35 (23.0%)
	Bilateral	13 (16.7%)	6 (8.1%)	NS	19 (12.5%)
# patients with fimbrioplasty (%)					
	Unilateral	11 (14.1%)	16 (21.6%)	NS	27 (17.8%)
	Bilateral	3 (3.8%)	9 (12.2%)	NS	12 (7.9%)
# patients with salpingostomy (%)					
	Unilateral	4 (5.9%)	3 (5.6%)	NS	7 (5.7%)
	Bilateral	0 (0.0%)	0 (0.0%)	NS	0 (0.0%)
# patients with salpingectomy (%)					
	Unilateral	0 (0.0%)	3 (4.2%)	NS	3 (2.0%)
	Bilateral	0 (0.0%)	0 (0.0%)	NS	0 (0.0%)
# patients with ovarian Cystectomy (%)					
	Unilateral	1 (1.3%)	6 (8.1%)	0.045	7 (4.6%)
	Bilateral	0 (0.0%)	0 (0.0%)	NS	0 (0.0%)
# patients with oophorectomy (#)					
	Unilateral	0 (0.0%)	1 (1.4%)	NS	1 (0.7%)
	Bilateral	0 (0.0%)	0 (0.0%)	NS	0 (0.0%)
# patients with ovarian suspension (#)					
	Unilateral	65 (85.3%)	71 (95.5%)	0.011	136 (89.5%)
	Bilateral	12 (15.4%)	3 (4.1%)	0.019	15 (9.9%)
# patients with tubes involved in endometriosis					
	Unilateral	23 (29.5%)	9 (12.2%)	0.009	32 (21.1%)
	Bilateral	11 (14.1%)	6 (8.1%)	NS	17 (11.2%)

In both groups there was no reported incidence of any major intra-operative complications such as excessive bleeding or late complications such as infection, hematoma or bowel herniation through the suture loop and its sequalae (bowel obstruction or strangulation). In 3 patients (3.4%) in Group 1 the suture was cut during securing of the knot on the fascia; all 3 cases occurred in the initial phase after the introduction of the technique. In these three patients slight bleeding occurred from the ovarian ligament which was controlled by bipolar coagulation using bipolar Kleppinger grasper (Wolf, Germany) and the suture was replaced. In one of these patients the suture was placed superficially, while in the other 2 patients it was deemed that excessive force was used during tying. In five patients (6.4%) in Group 1 and eight patients (10.8 %) in Group 2 suspension suture was deemed to be lax and not under tension at the end of the procedure. In all patients in both groups the ovary/ovaries were at approximately 5 cm from the abdominal wall at the conclusion of the procedure. One patient (1.3%) in Group 1 and one patient (1.4%) in Group 2 had post- operative retention of urine that required straight catheter, but all patients were discharged home same day of surgery. One patient (1.3%) in Group 1 and 2 patients (2.7%) in Group 2 developed urinary tract infection requiring oral antibiotics for one week. Post-operative pain, while in the hospital or after discharge was no different from other patients who underwent operative laparoscopy without ovarian suspension. All patients were discharged home on strong oral pain medication and had uneventful recoveries.

In patients in Group 1 the ovaries were found in its anatomical location on TV US that was done one week after surgery, while in Group 2 this was done 5-7 days post-operatively. Such finding may indirectly suggest that no adhesions occurred between the ovaries and the abdominal wall or the pelvic brim. No change in ovarian function was reported by any patient post-operatively or during the 2 years follow-up period, as judged by a change in menstrual cycle pattern. There was no evidence of decreased ovarian reserve post-operatively as determined by antral follicle count and serum day 3 FSH levels in patients who underwent COS + IUI or IVF-ET.

**Figure 3:**
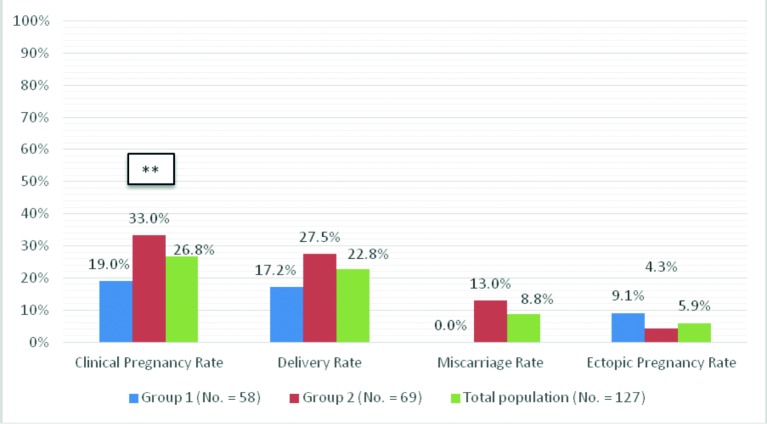
Pregnancy outcome after operative laparoscopy and ovarian suspension in patients who conceived with non- IVF methods after general exclusions and after excluding patients with severe male factor infertility.

**Figure 4:**
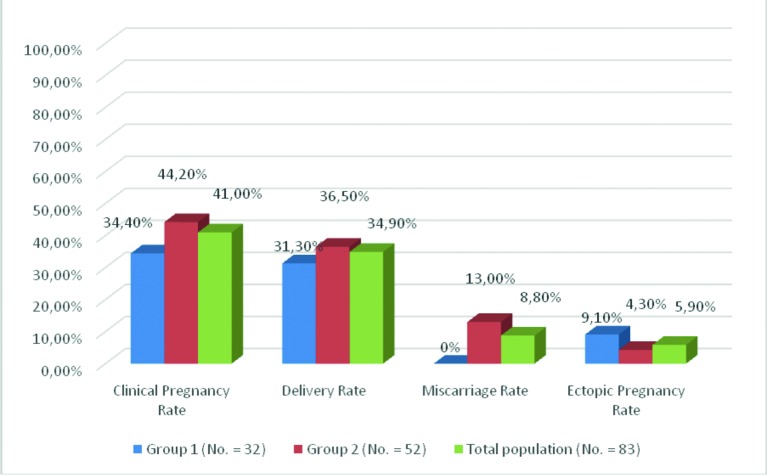
Pregnancy outcome after operative laparoscopy and ovarian suspension in patients who conceived with non- IVF methods after general exclusions and after excluding patients with severe male factor infertility and patients who conceived after IVF-ET.

Figure 3 illustrates pregnancy outcome of patients who conceived by non-IVF-ET methods after general exclusions and after exclusions of patients whose husbands had severe male factor infertility and opted not to have IVT-ICSI procedures done, as outlined in the Materials and Methods section. There was no significant difference between patients who conceived (19.0% vs 33.3%) delivered (17.2% vs 27.5%), miscarried (0.0% vs 13.0%), and had ectopic pregnancy (9.1% vs 4.3%) between Group 1 and Group 2. Figure 4 illustrates pregnancy outcome of patients who conceived by non-IVF-ET methods after excluding all patients who conceived by IVF-ET treatment, in addition, to the above-mentioned general exclusions criteria and after exclusion of patients with severe male factor infertility as outlined in materials and methods section. There was no significant difference in percentage of patients who conceived (34.4% vs 44.2%) delivered (31.3% vs 36.5%), miscarried (0.0% vs 13.0%), and had ectopic pregnancy (9.1% vs 4.3%) between Group 1 and Group 2 respectively.

## Discussion

This study describes the safety and efficacy of TOS to the anterior abdominal wall using absorbable and non-absorbable sutures after operative laparoscopy for advanced endometriosis (93.4%) and other indications (6.6%). It also compares the safety and efficacy of a modified technique using absorbable suture to the original technique published by Abuzeid M et al., (2002) using a non-absorbable suture (Abuzeid M et al., 2002; Abuzeid O et al., 2018). The results suggest that both techniques are simple, safe and easy to perform. Compared to the original technique published by Abuzeid M et al. (2002), the modified technique has the advantage of eliminating the theoretical risk of infection, eliminating the need for removal of the non-absorbable suture, and the additional benefit of suspending the ovary away from the pelvic brim and the fallopian tube, in turn reducing the risk of adhesions between the ovary and such structures (Figure 2) [Abuzeid M et al., 2002; Abuzeid O et al., 2018]. In addition, the modified technique allows one to leave fluids solutions in the peritoneal cavity for hydro-floatation. Such step was not utilized with the original technique to avoid soaking the gauze placed on the anterior abdominal wall, on which the non-absorbable suture was tied up, for fear of increasing the risk of infection (Abuzeid M et al., 2002). Therefore, during the last 7 years TOS using absorbable suture has been the preferred technique at our unit (Abuzeid O et al., 2018).

In both groups there was no reported incidence of any major intra-operative or post-operative complications such as injury of internal organs, excessive bleeding or infection. Similar results were reported by Seracchioli et al., (2014). A low complication rate was previously described by Poncelet et al., (2012). These authors observed two early complications, an ovarian abscess and hemoperitoneum, among 336 suspended ovaries (0.6%). In another study the authors observed one case of bowel strangulation (Hoo et al., 2014). In our study minor complications included urinary tract infection requiring oral antibiotics for one week and transient urinary retention requiring straight catheter. Post-operative analgesia was not different from what is normally prescribed after operative laparoscopy. Although infection was thought to be a potential risk after the use of non-absorbable 3-0 nylon suture as it was tied on a gauze and therefore in theory bacteria could spread to the peritoneal cavity along the suture while in place and during its removal; such complication was never reported in our current series of 74 patients (Group 2). To our knowledge infection was not reported by other authors who used a similar technique (Abuzeid M et al. 2002; Quahba et al., 2004; Mitwally et al., 2006). The modified technique described in Group 1 eliminates this theoretical risk (Abuzeid O et al., 2018). Bowel herniation through the suture loop and its sequalae (bowel obstruction or strangulation) was never observed in both groups. There were no other reports of such complication in the published literature except for one report by Hoo et al., (2014). We believe that if the technique described in Group 1 of this study and the original report by Abuzeid M et al., (2002), with respect to keeping the points of entry and exit of the suture through the fascia very close to each other and keeping the sutures under tension while placing the knot, is followed, such potential complication is very unlikely (Abuzeid M et al., 2002; Abuzeid O et al., 2018). Other minor complications that were described in the result section such as cutting the suture while tying the knot or tearing the ovarian ligament can be avoided by attention to details and being familiar with these potential complications. In brief, tearing the ovarian ligament while tying the knot on the fascia can be avoided by using a small tapered tip needle, taking a good bite of tissue, and by being delicate and gentle during that step.

The technique of TOS has been used for a variety of indications. It initially was used to protect the ovaries from the effects of radiation therapy by mobilizing the ovaries away from the radiation field (Lee et al., 1995, Paradisi et al., 2010). It was also used intra-operatively to facilitate surgical procedures during operative laparoscopy by lifting the ovaries away from the operative field (Cutner et al., 2004; Chapman et al., 2007; Chen et al., 2015).

It has also been used to try to reduce post-operative adhesion formation between the ovary and ovarian fossa after operative laparoscopy for advanced stages of endometriosis to help in relieving chronic pelvic pain and improving fertility potential (Redwine, 2001, Abuzeid M et al., 2002; Quahba et al., 2004; Mitwally et al., 2006; Carbonnel et al., 2011).

Various techniques have been proposed in the literature as to where the ovary should be suspended. Redwine, (2001) proposed TOS to the round ligament after operative laparoscopy for severe endometriosis using 3-0 Vicryl suture in patients with chronic pelvic pain secondary to severe endometriosis (Redwine, 2001). Similarly, Pellicano et al., (2014) reported that suspending the ovary to the ipsilateral round ligament using Vicryl Rapid 2.0 CT-1 needle after excision of an endometrioma resulted in significantly lower rate of postsurgical ovarian adhesion as judged by post- operative transvaginal outpatient hydro-laparoscopy (Pellicano et al., 2014). This technique of suspending the ovary to the round ligament, in theory, carries the risk of adhesion formation between these structures which certainly alters the normal anatomy and in turn may reduce fertility potential during natural attempts at achieving pregnancy. This may also make transvaginal oocyte retrieval during IVF difficult and potentially risky. Therefore, it seems that technique would be more suitable for patients who have completed their family and their main complaint is chronic pelvic pain. In contrast, the technique of TOS to the anterior abdominal wall that was initially proposed by Abuzeid M et al., (2002), and subsequently by Ouahba et al., (2004); Mitwally et al., (2006); Carbonnel et al., 2011 and Abuzeid O et al., (2018) is more suitable for patients who wish to preserve their reproductive potential. The technique allows the ovary to return to its normal anatomical location once the suture is removed.

The efficacy of the TOS technique can be best evaluated by second look laparoscopy. However, unfortunately second look laparoscopy is costly, may not be covered by insurance and not accepted by many patients. Therefore, there are only a few retrospective reports in the literature that describe the efficacy of these procedures based on a second look laparoscopy in small number of patients (Abuzeid M et al., 2002; Quahba et al., 2004; Carbonnel et al., 2011). All these studies and a systematic review of the literature by Pergialiotis et al., (2016) suggest that TOS to the anterior abdominal wall using non-absorbable sutures is an effective and feasible surgical technique, which might actually help reducing post-operative adhesions formation (Pergialiotis et al., 2016). Until recently the study by Pergialiotis et al., (2016) was the only systematic review of the literature on this topic which concluded that “current evidence suggests that ovarian suspension could be an effective and feasible surgical technique, which might actually help reduce postoperative adhesions”. However, a more recent systematic review of the literature by Giampaolino et al., 2018, reached the same conclusion.

There are two randomized prospective studies that evaluate post-operative adhesions formation after ovarian suspension based on non-invasive TV US findings with contradictory conclusions (Seracchioli et al., 2014; Hoo et al., 2014). These two studies used TV US for assessment of ovarian mobility as an indirect method of assessing post- operative adhesion formation. Although it remains controversial, several prospective studies suggested that TV US examination can be used as an alternative non-invasive approach that can accurately identify adhesions and ovarian mobility, with a good specificity and sensitivity of 90% and 89%, respectively (Guerriero et al., 1997; Holland et al., 2013). Seracchioli et al. (2014) suggested that TOS was associated with less adhesion development, but this was not associated with a reduction in pelvic pain, while Hoo’s et al., (2014) study suggested no benefit from TOS. Unfortunately, the latter study had a major flaw as the suture was removed after 36 hours which would be before peritoneal remesothelization is completed, and in turn this may have adversely affected the outcome of the procedure (Hoo et al., 2014; Trehan A and Trehan AK 2014). In our study we always evaluated the position of the ovaries by TV US after removal of the non-absorbable suture five to seven days after surgery in Group 2 and at the time of post-operative follow-up appointment one week after surgery in Group 1. In all patient, in both groups, the ovaries dropped to their anatomical position. Such findings in our study may indirectly suggest that TOS may be effective in reducing postoperative adhesion formation. Our findings are similar to the report by Seracchioli et al., (2014).

Another measure to assess the efficacy of TOS procedures is to report on pregnancy results post-operatively. In our study the percentage of patients who conceived after non-IVF methods may indirectly suggest that performing TOS at the conclusion of operative laparoscopy for advanced endometriosis may be beneficial in improving reproductive outcome. Other groups reported similar results (Quahba et al., 2004; Carbonnel et al., 2011). Unilateral TOS was performed in 83.3% in Group 1 and 95.9% in Group 2. This may influence the outcome of non-IVF methods, in the sense that pregnancy may have occurred from the side where TOS was not performed. Although that may be true, all patients with endometriosis (93.4%) had advanced disease and many had a pathology on the non-suspended side such as peri-tubal adhesions or a subtle fimbrial pathology that could reduce their chances of conception. In addition, a study published by Abuzeid M et al., (2009) suggested that pregnancy and delivery rates with non-IVF methods after operative laparoscopy for advanced stages of endometriosis were not different in patients with unilateral or bilateral adnexal involvement.

A thorough search of literature revealed that there are only 11 manuscripts and 1 published abstract addressing the safety and efficacy of TOS. There are 8 observational studies (7 manuscripts and 1 published abstract) [Abuzeid M et al., 2002; Quahba et al., 2004; Carbonnel et al. 2011; Poncelet et al., 2012; Pellicano et al., 2014; Trehan A and Trehan AK 2014; Abuzeid O et al., 2018, Mitwally et al., 2009], 2 systematic reviews (Pergialiotis et al., 2016; Giampaolino et al., 2018), and 2 prospective randomized control trials (Seracchioli et al., 2014; Hoo et al., 2014).

Our study has some limitation. First, our study is retrospective in nature with all the associated disadvantages of such design. We did not perform second look surgery, therefore, our results should be interpreted with caution regarding the efficacy of reducing post-operative adhesion formation. The data used to indirectly determine the efficacy, such as location of the ovaries on TV US and reproductive outcome, has its limitation. In addition, the study was done in one center with the senior author performing all procedures which may reduce the generalizability of the results. On the other hand, the study has its strength especially with respect to its large size and the fact that operative laparoscopy and both techniques of TOS in Group 1 and Group 2 were performed at one unit and by one surgeon.

## Conclusion

TOS to the fascia of the anterior abdominal wall using absorbable 3-0 plain catgut suture or non- absorbable mono filament nylon suture may help to displace the ovaries from the ovarian fossa during the time period of peritoneal repair. This may reduce postoperative adhesion development between the ovary and ovarian fossa. This procedure is simple, safe, easy to learn and has a potential effectiveness. TOS using absorbable suture is now our preferred technique. A prospective multicenter study, best with second look laparoscopy is required to confirm our findings.
